# Microstructure Evolution and Recrystallization Mechanisms of a Cu–Cr–Sn Alloy during Thermal Deformation Process

**DOI:** 10.3390/ma17092015

**Published:** 2024-04-25

**Authors:** Qian Yu, Zhen Yang, Lijun Peng, Haofeng Xie, Yicheng Cao, Yunqing Zhu, Feng Liu

**Affiliations:** 1 State Key Laboratory of Nonferrous Metals and Processes, GRIMN Group Co., Ltd., Beijing 100088, China; yuqian991227@163.com (Q.Y.); xiehaofeng@grinm.com (H.X.); caoyicheng1995@163.com (Y.C.); xz03080222@163.com (Y.Z.); 2 GRIMAT Engineering Institute Co., Ltd., Beijing 101407, China; 3 General Research Institute for Nonferrous Metals, Beijing 100088, China; 4 Ningbo Xingye Shengtai Group Co., Ltd., Ningbo 315336, China; liuf@cn-shine.com

**Keywords:** Cu–Cr–Sn alloy, thermal deformation behavior, constitutive model, microstructural evolution, softening mechanism

## Abstract

Thermal deformation behavior of Cu–Cr–Sn alloy ingots under deformation temperatures ranging from 600 °C to 950 °C and strain rates from 0.01 s^−1^ to 10 s^−1^ was investigated in detail. The thermal deformation constitutive equation and thermal processing map of the alloy were established, respectively. The activation energy Q was determined as 430.61 KJ/mol. The optimal deformation system corresponding to the hot working diagram was a deformation temperature of 900 °C and strain rate of 0.1 s^−1^. Under these deformation conditions, twin dynamic recrystallization (TDRX), continuous dynamic recrystallization (CDRX), and discontinuous dynamic recrystallization (DDRX) occurred simultaneously, with the twinning process causing the stress–strain curve to exhibit a wavy change. The thermal deformation microstructure of the alloy is co-regulated by different recrystallization mechanisms, with DDRX occurring mainly at low deformation temperatures, and both CDRX and DDRX occurring at high deformation temperatures.

## 1. Introduction

With the rapid development of the information age, integrated circuits play a crucial role in the modern industry. Lead frames, as key components in integrated circuits serving conductive, heat dissipation, and support functions, require good mechanical properties and excellent electrical conductivity [1,2,3]. Early lead frame materials were Fe–Ni alloys. Since the 1960s, the United States, Japan, Germany, and other countries have successively developed Cu–Fe, Cu–P, and Cu–Fe–P series alloys with better strength, electrical conductivity, and thermal conductivity to replace ferrous materials [4]. Since 2010, the development of very large-scale integrated circuits has prompted a surge in Cu–Ni–Si and Cu–Cr series alloys with higher strength and electrical conductivity. Currently, 80% of the world’s lead frames are made of copper alloy strips. Cu–Cr–Sn alloys, with their high strength and high conductivity characteristics, offer advantages such as ease of electroplating and good solderability compared to Cu–Cr–Zr and Cu–Ni–Si series alloys, meeting the application needs of very large-scale integrated circuits and making them ideal materials as high-end lead frames [5].

The thermal deformation process is usually accompanied by strain hardening processes dominated by dislocation movement and supplemented by twinning and softening processes led by dynamic recrystallization [6]. Both effects make it difficult for the alloy’s thermal deformation structure to form a strong texture. When large-scale Cu–Cr–Sn alloy ingots are present in industrial hot rolling production, cracking can easily occur. Therefore, it is necessary to determine a reasonable thermal processing regime and control the thermal deformation microstructure to improve production efficiency and material performance.

Recovery and recrystallization are extremely important mechanisms of microstructural evolution during the thermal deformation and are important ways to achieve microstructural softening, which play key roles in controlling the microstructures and properties of materials [7,8,9,10]. Dynamic recrystallization can be divided into discontinuous dynamic recrystallization and continuous dynamic recrystallization based on different mechanisms. Discontinuous dynamic recrystallization is the classic recrystallization that occurs through nucleation and growth, often initiated at grain boundaries bowing out, with growth achieved through the migration of high-angle grain boundaries [11,12,13]. During this process, the simultaneous formation and growth of dynamic recrystallization grains results in size differences and microstructural inhomogeneity between early- and later-formed grains. Continuous dynamic recrystallization does not involve a nucleation process; it mainly occurs through the absorption of dislocations by low-angle grain boundaries and subgrains merging to increase the orientation difference, ultimately forming high-angle grain boundaries. In this process, the substructure develops fully, and the orientation difference accumulates gradually, resulting in a uniform and fine microstructure.

The rheological behavior during the thermoplastic deformation process of the Cu–Cr–Sn alloy was described, and its softening modes under different thermal deformation conditions were also analyzed. In addition, the thermal processing map of the Cu–Cr–Sn alloy based on the dynamic materials model (DMM) [14] was established. The relationship between deformation temperature, strain rate, and the thermal deformation microstructure of the alloy was built, which provided, theoretically, guides for the hot rolling process of large-scale and wide Cu–Cr–Sn alloys.

## 2. Experimental Materials and Methods

### 2.1. Thermal Compression Experiment

The experimental material used in this paper was a large-scale Cu–Cr–Sn alloy ingot produced industrially. The alloy was melted in a 10-ton medium-frequency induction furnace using electrolytic copper, Cu–Cr master alloy, and pure tin as raw materials. The dimensions of the crystallizer were 620 mm in width × 190 mm in thickness × 230 mm in length. The chemical composition of Cu–Cr–Sn ingot is shown in Table 1.

**Figure 1:**
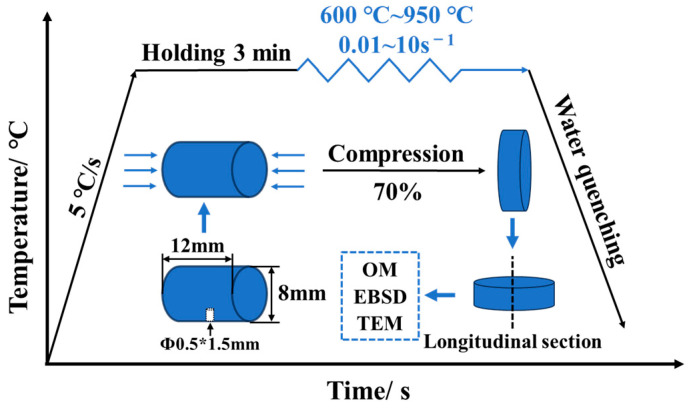
Schematic diagram of alloy’s thermal compression.

The ingot was processed into several cylindrical specimens with dimensions of Φ 8 mm × 12 mm. A small hole of Φ 0.5 mm × 1.5 mm was drilled at the center of the side of the cylinder to secure a thermocouple, thus preparing thermal compression samples for testing on a Gleeble-1500D thermal simulation testing machine. The selected deformation temperature range for the experiment was 600–950 °C, and the strain rate range was 0.01–10 s^−1^, with a total compressive true strain deformation of 70%. A schematic of the alloy’s thermal compression process is shown in Figure 1.

### 2.2. Microstructure Observation

The longitudinal sections of the thermally compressed samples were taken for metallographic observation, EBSD, and TEM characterization. The microstructure of the alloy was obtained using the Electron Backscatter Diffraction (EBSD) mode in the JEOL JSM-7900F scanning electron microscope. Electrolytic polishing was used to remove the stress layer from the surface of the finely ground samples. The recrystallization mode, grain size, grain orientation, and evolution of texture types of the alloy were analyzed using AZtecCrystal and OIM Analysis software. The microstructure and precipitate phases of the alloy were analyzed using a JEM-2010 high-resolution transmission electron microscope (TEM).

## 3. Experimental Results

### 3.1. True Stress–True Strain Curves

**Figure 2:**
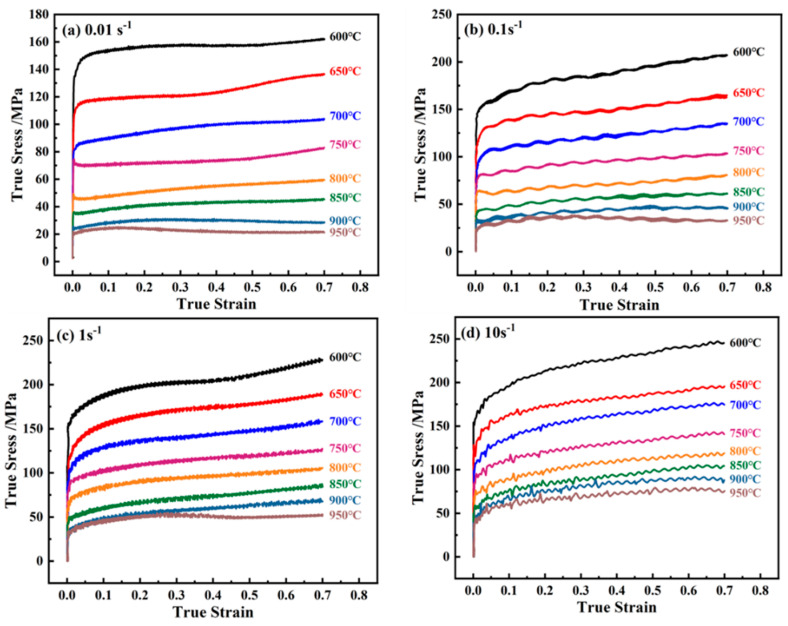
True stress–true strain curves of alloy at different strain rates: (**a**) $\overset{•}{\varepsilon }={0.01\;s}^{-1}$; (**b**) $\overset{•}{\varepsilon }={0.1\;s}^{-1}$; (**c**) $\overset{•}{\varepsilon }={1\;s}^{-1}$; and (**d**) $\overset{•}{\varepsilon }={10\;s}^{-1}$.

Figure 2 shows the true stress–true strain curves of Cu–Cr–Sn alloy at deformation temperatures ranging from 600 °C to 950 °C and strain rates from 0.01 s^−1^ to 10 s^−1^.

The peak stress of the alloy increases with the increase in strain rate and decreases with the increase in deformation temperature.

The alloy’s peak stress rises with increasing strain rate but declines with higher deformation temperatures. At low temperatures and strain rates (e.g., 600–800 °C and 0.01–0.1 s^−1^), as depicted in Figure 2a,b, the true stress–true strain curve stabilizes after a sharp stress increase during hardening, without a distinct peak, dominated by dynamic recovery. Under low temperatures and high strain rates (e.g., 600–800 °C and 1–10 s^−1^, as shown in Figure 2c,d), severe work hardening occurs, with continuous stress increase, dominated by hardening. At high temperatures and low strain rates (e.g., 800–950 °C and 0.01 s^−1^), stress initially rises then stabilizes, with peak stress decreasing as temperature rises. This occurs due to increased thermal activation of atoms, reducing lattice distortion and critical shear stress, facilitating dislocation movement. At high temperatures and high strain rates (e.g., 800–950 °C and 10 s^−1^, as shown in Figure 2c,d), the true stress–true strain curve displays wave-like variation, reaching dynamic equilibrium between work hardening and dynamic recrystallization softening.

### 3.2. Determination of Constitutive Equation and Its Parameters

The true stress–strain curve in Figure 2 can be used to obtain parameters such as flow stress, deformation temperature, and strain rate, which are utilized to solve for the thermal deformation constitutive equation and thermal processing map of the alloy.

The plastic deformation process of Cu–Cr–Sn alloy is a thermally activated process, in which the dislocation overcomes the resistance movement. The main macroscopic parameters of plastic deformation, such as deformation temperature, strain rate, and rheological stress, should follow the Arrhenius relation [15,16]:

(1) $$\overset{•}{\varepsilon }=A(\sigma )\exp(-Q/\mathrm{RT})$$

where Q is the deformation activation energy, and KJ/mol, which reflects the difficulty of deformation to a certain extent, is equivalent to the diffusion activation energy of the alloy. T is the thermodynamic temperature, K; R is the gas constant, R = 8.314 J/(mol·K).

For a given strain, A is only a function of stress A(σ); so, Equation (1) can be written as

(2) $$Z=\overset{•}{\varepsilon }\exp(+Q/\mathrm{RT})=A(\sigma )$$

Z is the Zener–Hollomon parameter, referred to as the Z-parameter. The Z-parameter is widely used to express the combined effect of deformation temperature and strain rate on thermal deformation [17]. The Z-parameter decreases with the increase in deformation temperature and the decrease in strain rate, and the Z-parameter is expressed differently at different stress levels [18,19,20,21]:

(3) $$\overset{•}{\varepsilon }{=A}_{1}\sigma^{n_{1}}\exp(-Q/\mathrm{RT})\;\left(\alpha \sigma <0.8\right)$$

(4) $$\overset{•}{\varepsilon }{=A}_{2}\exp(\beta \sigma )\exp(-Q/\mathrm{RT})\;\left(\alpha \sigma >1.2\right)$$

(5) $$\overset{•}{\varepsilon }{=A[\sin h(\alpha \sigma )]}^{n}\exp(-Q/\mathrm{RT})\;\left(\mathrm{all}\sigma \right)$$

**Figure 3:**
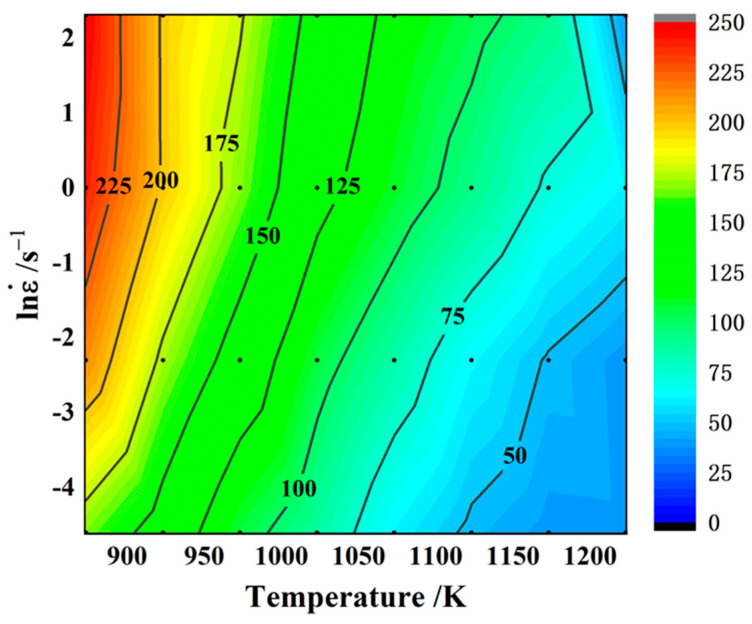
Peak stress contour plots of alloys under different deformation conditions.

A_1_, n_1_, A_2_, β, A, α, and n are all material constants, and α = β/n_1_. For the σ in Equations (3)–(5), the peak stress, the steady-state rheological stress, and the instantaneous rheological stress corresponding to a certain strain variable can be expressed, and the following derivation process σ specifically refers to the peak stress σ_p_. Figure 3 is the peak stress contour diagram of Cu–Cr–Sn alloys under different deformation conditions.

Take the logarithm on both sides of Equations (3)–(5) as follows:

(6) $$\ln\overset{•}{\varepsilon }{=n}_{1}{\ln A}_{1}-Q/\mathrm{RT}$$

(7) $$\ln\overset{•}{\varepsilon }{=\beta \sigma +\ln A}_{2}-Q/\mathrm{RT}$$

(8) $$\ln\overset{•}{\varepsilon }=\mathrm{nln}[\sin h(\alpha \sigma )]-Q/\mathrm{RT}+\ln A$$

**Figure 4:**
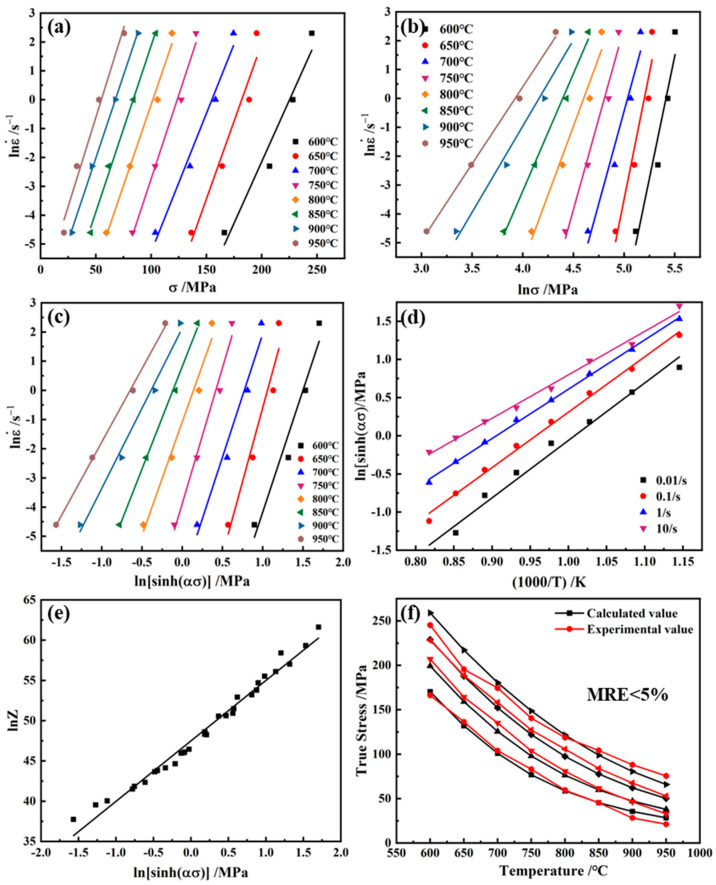
Relationship images between (**a**) $\ln\overset{•}{\varepsilon }-\ln\sigma$; (**b**) $\ln\overset{•}{\varepsilon }-\sigma$; (**c**) $\ln\overset{•}{\varepsilon }-\ln[\sin h(\alpha \sigma )]$; (**d**) ln[sinh(ασ)]−(1/1000T); (**e**) lnZ−ln[sinh(ασ)]; and (**f**) calculated–experimental true stress values.

Figure 4a,b were made with $\ln\overset{•}{\varepsilon }-\ln\sigma$ and $\ln\overset{•}{\varepsilon }-\sigma$ as coordinates, respectively, and linear regression was carried out by the least squares method. The parameters $n_{1}=\frac{\partial \ln\overset{•}{\varepsilon }}{\partial \ln\sigma }=11.11$ and $\beta =\frac{\partial \ln\overset{•}{\varepsilon }}{\partial \sigma }=0.11$ of the constitutive equation could be obtained from the slope, and the parameters ${\alpha =\beta /n}_{1}=0.0098$ could be further solved.

For Equation (8), assuming that the deformation activation energy is independent of temperature, when the deformation temperature is constant, the partial derivative of the strain rate on both sides is calculated at the same time, and Figure 4c is made with $\ln\overset{•}{\varepsilon }-\ln[\sin h(\alpha \sigma )]$ as the coordinate. The linear regression is performed by the least squares method, and the constitutive equation parameter $n=\frac{\partial \ln\overset{•}{\varepsilon }}{\partial \ln[\sin h(\alpha \sigma )]}=7.69$ can be obtained from the slope. When the strain rate is constant, the partial derivative of 1/1000T is found on both sides at the same time, and the ln[sinh(ασ)]−(1/1000T) is used as in Figure 4d. The least squares method is used for linear regression, and $b=\frac{\partial \ln[\sin h(\alpha \sigma )]}{\partial \left(1/1000T\right)}=6.74$, is obtained from the slope, then the deformation activation energy of the alloy is Q=Rnb=430.61 KJ/mol.

At all stress levels, the Z-parameter can be expressed as

(9) $${Z=A[\sin h(\alpha \sigma )]}^{n}$$

Take the logarithm on both sides of the equation, as seen below.

(10) lnZ=lnA+nln[sinh(ασ)]

The constitutive equation of the Cu–Cr–Sn alloy can be expressed as

(11) $$\overset{•}{\varepsilon }{=e}^{47.49}{\left[\sin h(0.0098\sigma )\right]}^{7.69}\exp(\frac{-430.61}{\mathrm{RT}})$$

The stress values calculated by the constitutive equation are compared with the peak stress values, as shown in Figure 4f, with a relative average error of less than 5%.

### 3.3. Establishment of Hot Working Map

The establishment of a reasonable thermal processing diagram is an important means to guide the thermal processing system of alloys. At present, the dynamic material model (DMM) has been widely used in the drawing of thermal process drawings. In the DMM, the alloy itself can be used as an energy-dissipating system, and for a given deformation condition, the total power dissipated P per unit volume of the alloy during thermoplastic deformation consists of two parts, G and J [9,22]:

(12) $$P=\sigma \overset{•}{\varepsilon }=G+J=\int_{0}^{\overset{•}{\varepsilon }}\sigma d\overset{•}{\varepsilon }+\int_{0}^{\sigma }\overset{•}{\varepsilon }d\sigma$$

where G is the power dissipation caused by the deformation process, and J is the power dissipation caused by the evolution of the microstructure. At the same time, under different deformation conditions, the effect of the strain rate on the flow stress can be expressed as

(13) $$\sigma =C{\overset{•}{\varepsilon }}^{m}$$

where C is a constant depending on strain, temperature, and strength of the material, and m is the strain rate sensitivity index. Due to the acceleration of thermal activation processes such as dislocation motion and grain boundary slip after increasing temperature, the value m usually increases with the increase in deformation temperature. The value m can be found from the ratio of G and J:

(14) $$m={\left(\frac{\partial \ln\sigma }{\partial \ln\overset{•}{\varepsilon }}\right)}_{\varepsilon ,T}$$

From this, Equation (12) can be expressed as

(15) $$P=G+J=\int_{0}^{\overset{•}{\varepsilon }}C{\overset{•}{\varepsilon }}^{m}d\overset{•}{\varepsilon }+\int_{0}^{\sigma }{\left(\sigma /C\right)}^{1/m}d\sigma =\frac{\sigma \overset{•}{\varepsilon }}{m+1}+\frac{\sigma \overset{•}{\varepsilon m}}{m+1}$$

For an ideal linear dissipative workpiece, J reaches a maximum value at m = 1:

(16) $$J_{\max}=\frac{1}{2}\sigma \overset{•}{\varepsilon }=\frac{P}{2}$$

Then, the power dissipation efficiency η can be expressed as

(17) $$\eta =\frac{J}{J_{\max}}=\frac{2J}{P}=\frac{2m}{m+1}$$

When the strain variable is 70%, the power dissipation efficiency η contour plots at different deformation temperatures and strain rates are plotted as the power dissipation plots of the alloy. Generally, the higher the power dissipation efficiency of the alloy, the better the hot workability of the corresponding alloy. However, the unstable factors, such as adiabatic shear bands and cracks that may exist in the alloy processing process, lead to increase the power dissipation efficiency of the alloy. Therefore, the continuous instability criterion of Equation (18) is used as the basis for the division of rheological instability regions in the process of thermal deformation:

(18) $$\xi (\overset{•}{\varepsilon })=\frac{\partial \ln\left(\frac{m}{m+1}\right)}{\partial \ln\overset{•}{\varepsilon }}+m<0$$

**Figure 5:**
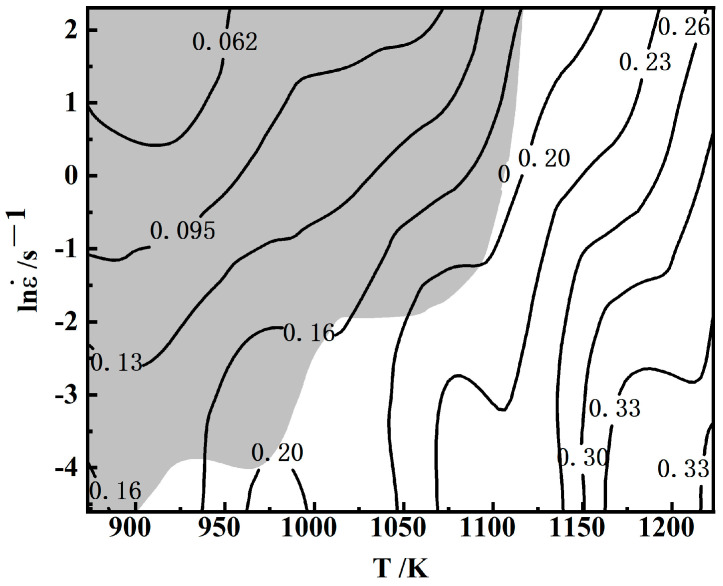
Thermal working diagram of Cu–Cr–Sn alloy.

The region of ξ < 0 is divided into rheological instability regions, which are represented by gray color blocks. The thermal working diagram of the Cu–Cr–Sn alloy is obtained by plotting the instability map under the same deformation conditions and superimposing this part of the area on the power dissipation map, as shown in Figure 5.

The thermal processing diagram of the alloy at a deformation level of 70% can be divided into two parts: at high deformation temperatures and low strain rates, it mainly represents the processing safety zone; while at low deformation temperatures and high strain rates, it primarily indicates the rheological instability zone. It can be observed that with increasing deformation temperature and decreasing strain rate, the power dissipation rate of the alloy gradually increases. Below 750 °C, the corresponding zones for different strain rates are all unstable zones. At 750 °C, the alloy falls into the safety zone when the strain rate is 0.01 s^−1^ or 0.1 s^−1^; however, it enters the unstable zone at strain rates of 1 s^−1^ or 10 s^−1^. At a deformation temperature of 900 °C and strain rates of 0.01 s^−1^ or 0.1 s^−1^, the power dissipation rate of the alloy reaches its maximum at 33%. Within the range of 850 to 950 °C, at strain rates of 0.01 s^−1^ or 0.1 s^−1^, the power dissipation rate of the alloy exceeds 30%, indicating suitable processing conditions.

### 3.4. Microstructural Evolution

#### 3.4.1. As-Cast Microstructure of Cu–Cr–Sn Alloy

**Figure 6:**
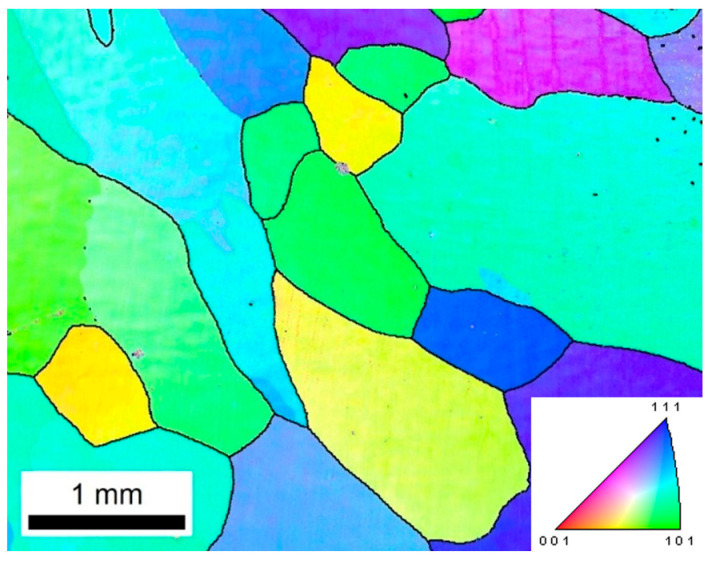
The inverse pole figure of Cu–Cr–Sn alloy ingot microstructure.

The as-cast microstructure of the large-scale Cu–Cr–Sn alloy ingot exhibits larger equiaxed grains, which show the random orientation distribution, as shown in Figure 6.

#### 3.4.2. EBSD Analysis under Different Deformation Conditions

**Figure 7:**
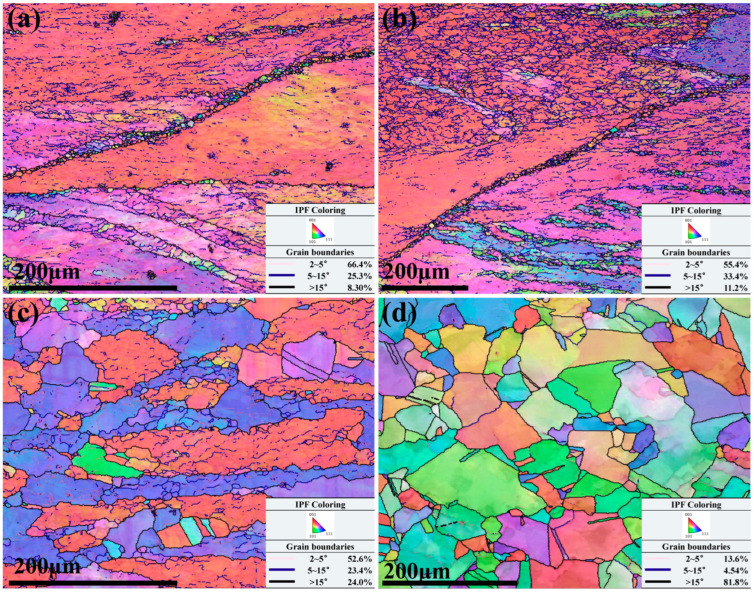
IPF maps of Cu–Cr–Sn alloy at a strain rate of 0.01 s^−1^ with (**a**) deformation temperature of 750 °C; (**b**) deformation temperature of 800 °C; (**c**) deformation temperature of 850 °C; and (**d**) deformation temperature of 900 °C.

The IPF diagrams of the Cu–Cr–Sn alloy at different temperatures for strain rate at 0.01 s^−1^ are shown in Figure 7.

It can be seen from Figure 7a,b, some small-sized recrystallized grains with orientations different from the deformed grains are found along the grain boundaries when the deformation temperature is 750 °C and 800 °C, respectively. As the deformation temperature increases to 850 °C, the broken deformed grain boundaries have a serrated feature, and many new recrystallized grains are generated along the grain boundaries, and the average size of recrystallized grains has grown to 75 μm [12]. In addition, the orientation of grains is observed along the [111]_Cu_ orientation other than [011]_Cu_. The deformation temperature is 900 °C, and a large number of grains are found along the [101]_Cu_ orientation, which is consistent with the earliest glide direction of face-centered cubic structure metals such as the Cu–Cr–Sn alloy, as shown in Figure 7d. It indicates that the grains begin to rotate as the deformation temperature increases.

Under the same strain conditions, as the deformation temperature increases, the thermal activation is enhanced, and the grain boundary migration rate accelerates, which is conducive to the migration of new recrystallized grains out of the serrated grain boundaries of the original grains and their continued growth. The proportion of 2–5° subgrain boundaries gradually decreases, transforming into low-angle grain boundaries or even high-angle grain boundaries, and the proportion of high-angle grain boundaries greater than 15° gradually increases.

**Figure 8:**
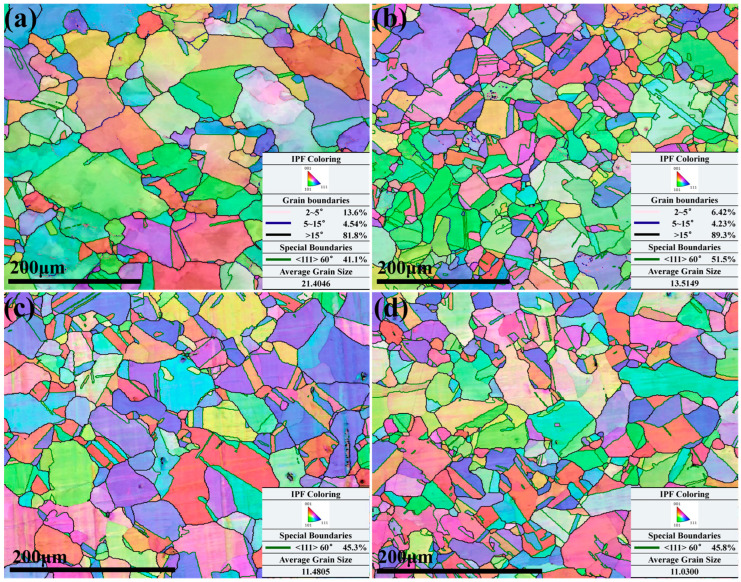
IPF maps of Cu–Cr–Sn alloy at a deformation temperature of 900 °C and (**a**) strain rate of 0.01 s^−1^; (**b**) strain rate of 0.1 s^−1^; (**c**) strain rate of 1 s^−1^; and (**d**) strain rate of 10 s^−1^.

The microstructure of the Cu–Cr–Sn alloy corresponding to a deformation temperature of 900 °C and different strain rates is shown in Figure 8.

The recrystallization behavior is obviously found during the deformation, and nuclei were preferentially located at the areas with high local strain. When the deformation amount exceeds a critical value, the nucleation rate increases sharply with the increase in deformation amount. As the strain rate decreases, the driving force for alloy increases, which contributes to accelerate the migration rate of grain boundaries and separate new grains from the original grains. At a strain rate of 0.01 s^−1^, the average grain size of the Cu–Cr–Sn alloy is about 21 μm, as shown in Figure 8a. When the strain rate increases to 0.1 s^−1^, the average grain size decreases to 13 μm. The strain rate is 1 s^−1^ and 10 s^−1^, respectively, and the average grain size has no significant change, as shown in Figure 8c,d. It indicates that at lower strain rates, the grain size is larger, which may be due to the higher accumulated strain energy. Therefore, there is still residual energy driving the growth of recrystallized grains after recrystallization nucleation. At higher strain rates, many twin boundaries appear to assist subgrain co-deformation. In the newly formed recrystallized grains, both recrystallized grains at the tens of micrometer scale and recrystallized grains at the micron scale are found. It can be inferred that the larger recrystallized grains are mainly produced by subgrain rotation in the original deformed structure, while the smaller recrystallized grains may be generated by the bowing out of serrated grain boundaries and limited growth by surrounding recrystallized grains. It may be formed at high-energy locations of the tricrystal boundaries in the new recrystallized grains due to higher strain energy driving. Higher strain rates can promote the nucleation of new grains by increasing the stored energy and creating more favorable conditions for nucleation sites to form. These things considered, higher strain rates can also affect the mobility of grain boundaries, promoting their migration and facilitating grain boundary motion required for recrystallization. The early-formed recrystallized grains grow rapidly, which hinders the growth of adjacent grains, resulting in uneven grain size. The twin boundaries in the microstructure under different strain rates in Figure 8 are marked with green lines. It can be seen that proportion of twins increases with the increase in strain rate deformation at 900 °C. However, the proportion of twins is the highest at a strain rate of 0.1 s^−1^, reaching 51.5%, as shown in Figure 8b. This may be because merging and rotation of small-angle grain boundary and twinning are simultaneously produced during the plastic deformation process.

**Figure 9:**
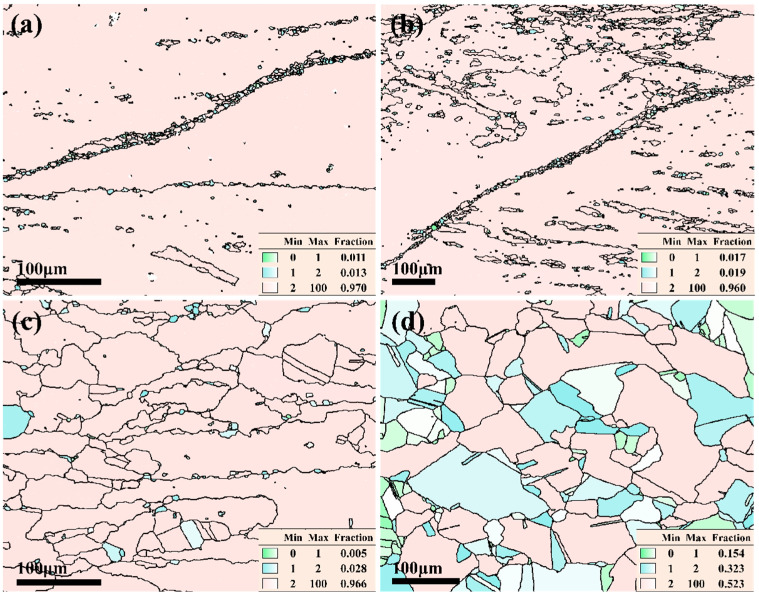
Grain orientation spread maps of Cu–Cr–Sn alloy at a strain rate of 0.01 s^−1^ with (**a**) deformation temperature of 750 °C; (**b**) deformation temperature of 800 °C; (**c**) deformation temperature of 850 °C; and (**d**) deformation temperature of 900 °C.

**Figure 10:**
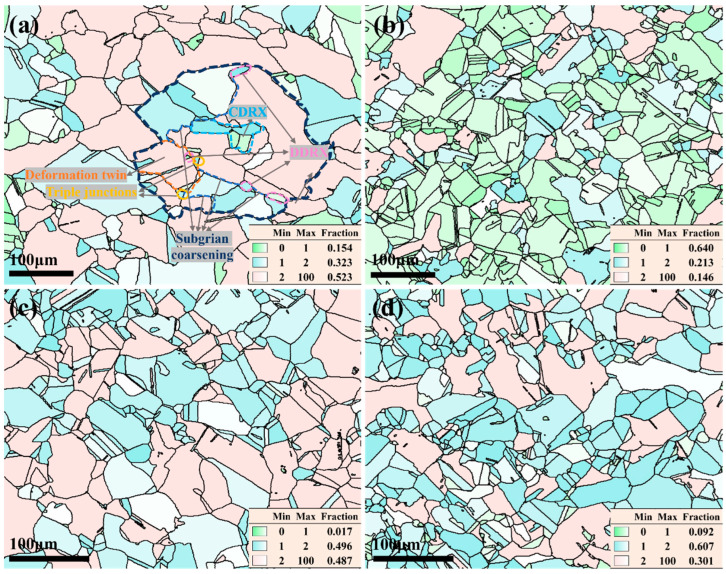
Grain orientation spread maps of Cu–Cr–Sn alloy at a deformation temperature of 900 °C with (**a**) strain rate of 0.01 s^−1^; (**b**) strain rate of 0.1 s^−1^; (**c**) strain rate of 1 s^−1^; and (**d**) strain rate of 10 s^−1^.

Figure 9 and Figure 10 are grain orientation spread maps within the alloy grains, respectively, where 0–1° stands for fully recrystallized structures, 1–2° stands for partially recrystallized structures, and greater than 2° corresponds to deformed structures.

The black lines stand for high-angle grain boundaries in Figure 9. Many deformed structures are found, and a few of the recrystallized structures are also observed along the boundary of the deformed structure when the deformation temperature is at 750 °C, 800 °C, and 850 °C for a strain rate of 0.01 s^−1^, as seen in Figure 9a–c.

From 750 °C to 800 °C, subgrains within the deformed grains gradually transform into high-angle grain boundaries, and a certain proportion of high-angle grain boundaries appear inside the deformed grains. As the deformation temperature rises to 850 °C, the speed of grain boundary migration accelerates, resulting in increasing smaller-sized deformed structural grains. However, the grain boundaries of these newly formed deformed grains are restricted by the orientation of the subgrains. The grain boundaries are not completely straight, exhibiting serrated characteristics. Therefore, some smaller new recrystallized nuclei appear along the grain boundaries. This is mainly because the substructure density on both sides of the grain boundaries of the newly formed deformed grains is inconsistent, with the side of higher substructure density bowing out towards the side of lower density, making the grain boundaries tend to straighten. Due to the bowing out of grain boundaries, new recrystallized nuclei are formed. Additionally, the energy of triple junctions is higher and unstable, which also serves as a preferential site for recrystallization nucleation. When the deformation temperature is 900 °C, 32.3% of partially recrystallized grains and 15.4% of fully recrystallized grains are found with the grains that underwent complete recrystallization generally being smaller in size. Combined with the IPF map in Figure 6, the deformed structures are still basically found corresponding to the [001] orientation in the IPF map, while recrystallization mainly occurs along the [101] orientation, and some occur along the [111] orientation.

The hot deformation structure under the conditions of a deformation temperature of 900 °C and a strain rate of 0.01 s^−1^ is shown in Figure 10a, and various mechanisms for recrystallization nucleation are obviously observed. The pink lines represent smaller recrystallized grains that appear at the boundaries of the original deformed grains, generated by boundary migration, belonging to discontinuous dynamic recrystallization [23] (DDRX); the blue lines are concentrated within the original deformed grains, formed by the rotation and coalescence of subgrain boundaries inside the grains to create new recrystallized grains, belonging to continuous dynamic recrystallization (CDRX); and the yellow lines represent the triple junctions of highly deformed grains, where the grain boundaries are higher, serving as nucleation points for recrystallization. The proportion of fully recrystallized grains is highest with uniformly fine grain sizes, including partially recrystallized grains, while only a small number of deformed grains are retained when the Cu–Cr–Sn alloy deformed for 900 °C at a strain rate of 0.1 s^−1^, as seen in Figure 10b. As the strain rate increases to 10 s^−1^, a high proportion of recrystallization is also found.

Considering the uniformity of the alloy structure and processing efficiency, the reasonable processing system can be confirmed, e.g., deformation temperature of 900 °C and a strain rate of 0.1 s^−1^. At these conditions, the power dissipation value in the thermal processing map of the corresponding alloy reaches its maximum, indicating efficient energy utilization during processing.

**Figure 11:**
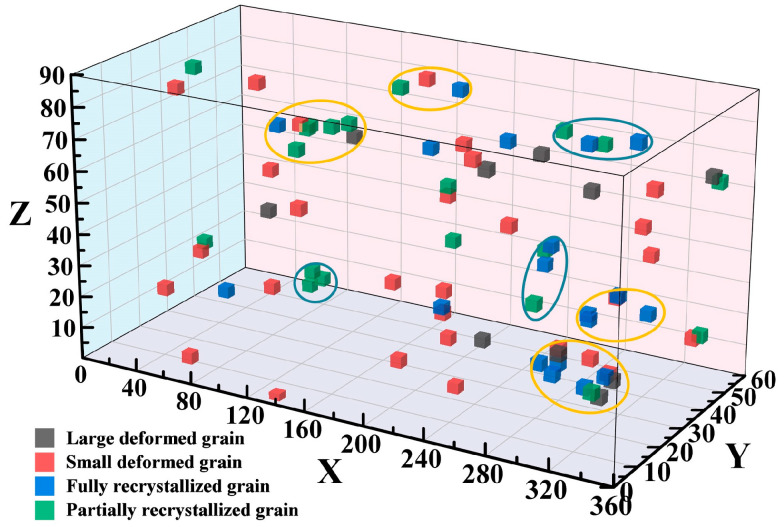
Orientation spatial distribution map of grains at a strain rate of 0.01 s^−1^ under 900 °C.

According to Figure 10a, the orientation relationships among fully recrystallized grains, partially recrystallized grains, and original deformed grains are investigated in detail, as shown in Figure 11.

Gray cubes indicate large deformed grains, red cubes indicate small deformed grains, blue cubes indicate fully recrystallized grains, and green cubes indicate partially recrystallized grains, respectively. It can be observed that the orientation distribution of the small deformed grains is relatively random, and there are two main types of orientation distribution relationships between the fully recrystallized grains, partially recrystallized grains, and the deformed grains. One is as shown in the yellow circles, where the orientation of the recrystallized grains essentially remains consistent with that of the deformed grains, corresponding to DDRX in Figure 10a. The other is as shown in the blue circles, where new isolated orientations of recrystallized grains appear, corresponding to CDRX in Figure 10a. This indicates that under high deformation temperature and low strain rate, dynamic recrystallization is mainly dominated by DDRX, while some grains undergo CDRX.

#### 3.4.3. TEM Observation

**Figure 12:**
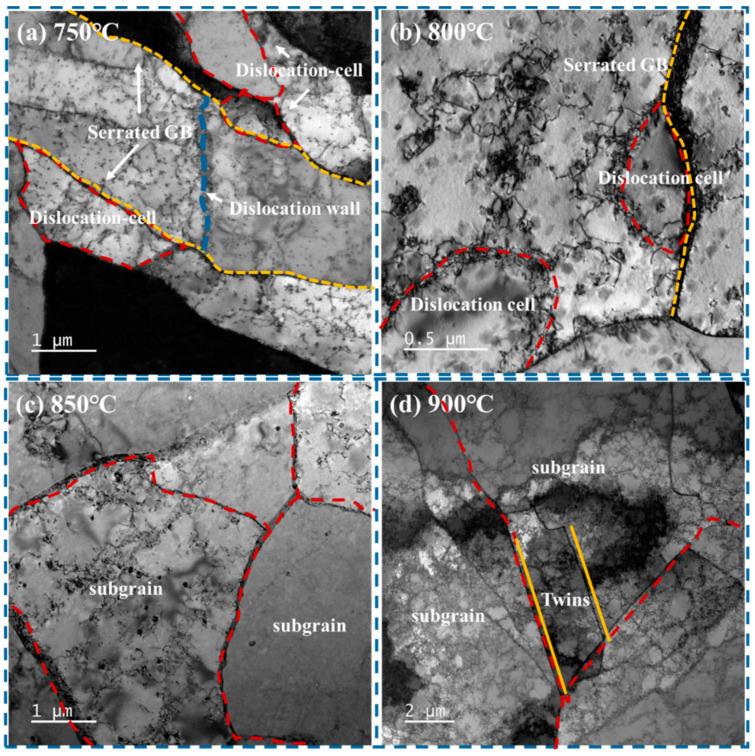
TEM microstructure of Cu–Cr–Sn alloy at deformation temperatures of (**a**) 750 °C, (**b**) 800 °C, (**c**) 850 °C, and (**d**) 900 °C.

The TEM observation of the Cu–Cr–Sn alloy corresponding to deformation temperatures of 750 °C, 800 °C, 850 °C, and 900 °C at strain rate is shown in Figure 12.

It can be seen from Figure 12a,b that fine new recrystallized grains appear along serrated grain boundaries at low deformation temperatures, and a significant amount of dislocation substructures still exist in the deformed grains, which shows a typical characteristic of discontinuous dynamic recrystallization (DDRX) [24,25,26,27]. This is in accordance with Figure 9a,b. With deformation temperature increasing, the intragranular dislocation density decreases, subgrain coalescence and rotation occur, and new large-angle grain boundaries are found, which are predominantly characterized by continuous dynamic recrystallization (CDRX) [28,29,30], as shown in Figure 12c. This is consistent with Figure 9c. In Figure 12d, in addition to changes in dislocation substructure, twins are produced at 900 °C, which corresponds to Figure 10a,b. This is consistent with Figure 9c,d.

## 4. Discussion

**Figure 13:**
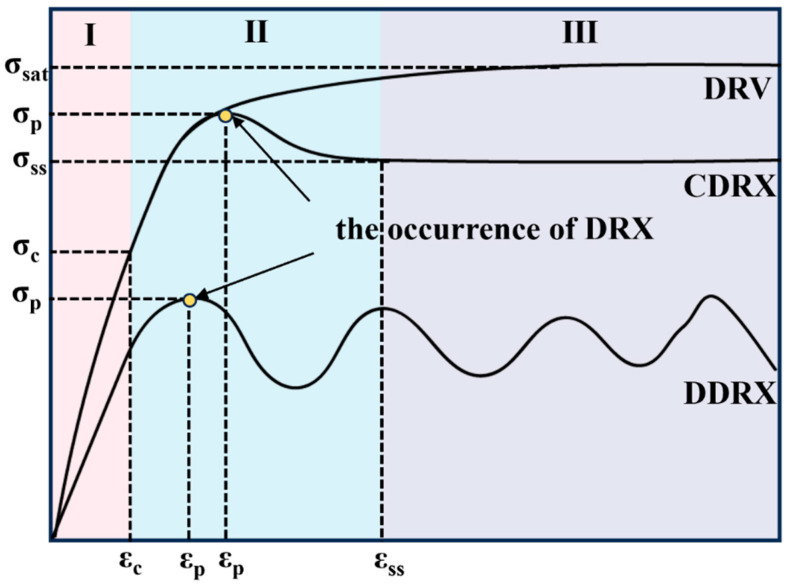
Schematic diagram of the stress–strain characteristic curve for thermoplastic deformation.

Figure 13 shows a schematic diagram of the stress–strain characteristic curve for thermoplastic deformation.

Stage I is the work hardening stage, the alloy undergoes softening in stage II, and in stage III, the alloy’s hardening and softening reach dynamic equilibrium. The curve DRV represents the stress–strain characteristic curve of dynamic recovery (DRV), which is similar to the curve of the Cu–Cr–Sn alloy deformed at 650–800 °C for all strain rates, as shown in Figure 2. Curve CDRX depicts deformation behavior under higher strain rates and lower deformation temperatures, which can be typically divided into three stages: The first stage is the work hardening stage before dynamic recrystallization occurs, serving as the preparatory phase for inducing dynamic recrystallization. The second stage is the partial dynamic recrystallization stage where the strain reaches the critical amount of deformation required for dynamic recrystallization to occur. As the strain increases, the slope of the curve decreases, and after the strain rises to its maximum value, the curve declines, indicating that dynamic recrystallization is intensifying. The third stage is the complete dynamic recrystallization stage where work hardening and recrystallization softening reach equilibrium, the curve levels off, and the flow stress approaches a constant value, achieving stable deformation, typically corresponding to continuous dynamic recrystallization (CDRX). The characteristic of curve CDRX is similar to the curve of the Cu–Cr–Sn alloy deformed at 900–950 °C for strain rate 0.01 s^−1^ and 1 s^−1^, as shown in Figure 2a,c. Curve III represents deformation behavior under low strain rates and higher deformation temperatures where the rate of increase in dislocation density is small. After dynamic recrystallization, further work hardening is required to accumulate dislocation density for recrystallization to occur again, resulting in a wavy curve. The feature of curve DDRX is similar to the curve of the Cu–Cr–Sn alloy deformed at 900–950 °C for strain rate 0.1 s^−1^, as shown in Figure 2b. This is the outcome of the repeated occurrence of dynamic recrystallization–deformation–dynamic recrystallization, a cycle of softening and hardening, typically corresponding to discontinuous dynamic recrystallization.

**Figure 14:**
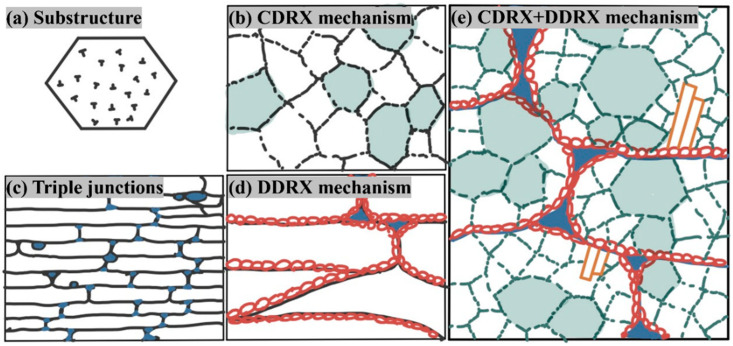
The schematic diagram of the evolution of the recrystallized organization during the hot deformation process. (**a**) Substructure; (**b**) CDRX mechanism; (**c**) triple junctions; (**d**) DDRX mechanism; and (**e**) CDRX+DDRX mechanism.

Figure 14 shows the schematic diagram of the evolution of the recrystallized organization during the hot deformation process.

The nucleation of discontinuous dynamic recrystallization usually starts at the initial grain boundaries, as shown in Figure 12a. When there is a large difference between the initial grain size and the recrystallized grain size, a necklace-like structure consisting of equiaxed grains is formed, as shown in Figure 7a,b. The recrystallization speed increases with the decreases in initial grain size, strain rate, and deformation temperature. During discontinuous dynamic recrystallization, the grain size increases towards the saturation value and then remains constant as recrystallization continues, as shown in Figure 10a.

The occurrence of continuous dynamic recrystallization begins at the recovery stage, during which subgrain boundaries are formed inside the polycrystal. The misorientation angle of subgrain boundaries is only a few degrees, which is much lower than that of high-angle grain boundaries.

Throughout the deformation process in Figure 8 and Figure 12c, mobile dislocations continually enter the subgrain boundaries, and the lattice dislocations on low-angle subgrain boundaries trap these mobile dislocations, leading to an increase in misorientation. Thus, the subgrain boundaries gradually transform into high-angle grain boundaries, meaning the subgrains will eventually transform into recrystallized grains.

The average grain size of recrystallization is negatively correlated with the degree of deformation, and after the strain increases to a certain extent, it reaches a stable value, and some stable original grains remain unchanged even at high strains. Under large strain deformation, reducing the initial grain size can significantly accelerate grain refinement.

## 5. Conclusions

Hot deformation behavior of the Cu–Cr–Sn alloy was investigated under temperatures of 650–900 °C and strain rates of 0.01–10 s^−1^. Based on the obtained experimental and modeling results, the following conclusions can be drawn:

1. The activation energy of the alloy was calculated as 430.61 KJ/mol, and the constitutive equation of the Cu–Cr–Sn alloy can be represented as $\overset{•}{\varepsilon }{=e}^{47.49}{\left[\sin h(0.0098\sigma )\right]}^{7.69}\exp(\frac{-430.61}{\mathrm{RT}})$.
2. Thermal processing map of the alloy was established, and the reasonable thermal processing system for the Cu–Cr–Sn alloy was a deformation temperature of 900 °C and a strain rate of 0.1 s^−1^.
3. The DDRX was mainly dominant at low deformation temperatures, while CDRX and DDRX both happened at high deformation temperatures. At a strain rate of 0.1 s^−1^, the proportion of twinning was the highest, and twinning nucleation was the main nucleation mode for recrystallization.

## Figures and Tables

**Table 1:** Chemical composition of the designed alloy.

Alloy	Alloy Element (wt.%)		
	Cr	Sn	Cu
Cu–Cr–Sn	0.22	0.2	Bal.

## Data Availability

Data is unavailable due to privacy.
